# 100 % Fruit juice and measures of glucose control and insulin sensitivity: a systematic review and meta-analysis of randomised controlled trials

**DOI:** 10.1017/jns.2017.63

**Published:** 2017-12-15

**Authors:** Mary M. Murphy, Erin C. Barrett, Kara A. Bresnahan, Leila M. Barraj

**Affiliations:** 1 Exponent, Inc., Center for Chemical Regulation & Food Safety, 1150 Connecticut Avenue, NW, Washington, DC 20036, USA; 2 Habit, LLC, 985 3rd Street, Oakland, CA 94607, USA

**Keywords:** 100 % Fruit juice, Fasting blood glucose, Fasting blood insulin, Insulin sensitivity, Homeostatic model assessment of insulin resistance, HbA1c, glycosylated Hb, HOMA-IR, homeostatic model assessment of insulin resistance, RCT, randomised controlled trial, T2D, type 2 diabetes

## Abstract

Studies on the effects of consuming 100 % fruit juice on measures of glycaemic control are conflicting. The purpose of the present study was to systematically review and quantitatively summarise results from randomised controlled trials (RCT) examining effects of 100 % fruit juice on glucose–insulin homeostasis. Eligible studies were identified from a systematic review of PubMed and EMBASE and hand searches of reference lists from reviews and relevant papers. Using data from eighteen RCT, meta-analyses evaluated the mean difference in fasting blood glucose (sixteen studies), fasting blood insulin (eleven studies), the homeostatic model assessment of insulin resistance (HOMA-IR; seven studies) and glycosylated Hb (HbA1c; three studies) between the 100 % fruit juice intervention and control groups using a random-effects model. Compared with the control group, 100 % fruit juice had no significant effect on fasting blood glucose (−0·13 (95 % CI −0·28, 0·01) mmol/l; *P* = 0·07), fasting blood insulin (−0·24 (95 % CI −3·54, 3·05) pmol/l; *P* = 0·89), HOMA-IR (−0·22 (95 % CI −0·50, 0·06); *P* = 0·13) or HbA1c (−0·001 (95 % CI −0·38, 0·38) %; *P* = 0·28). Results from stratified analyses and univariate meta-regressions also largely showed no significant associations between 100 % fruit juice and the measures of glucose control. Overall, findings from this meta-analysis of RCT suggest a neutral effect of 100 % fruit juice on glycaemic control. These findings are consistent with findings from some observational studies suggesting that consumption of 100 % fruit juice is not associated with increased risk of diabetes.

Worldwide, the number of people with diabetes is rising. In 2014, the estimated prevalence among adults was 8·5 %, which is approximately double the prevalence of 4·7 % in 1980^(^^1^^)^. Due to its prevalence and associated complications, diabetes is a well-recognised public health concern^(^^2^^)^. Overweight and obesity are the strongest risk factors for type 2 diabetes (T2D), though lifestyle and dietary modification also are recognised strategies that may delay or prevent development of the disease.

Controversy and uncertainty have been expressed within the scientific community as to what effect, if any, 100 % fruit juice may have on health including risk for diabetes^(^^3^^)^. Pure (100 %) fruit juices can be nutrient-dense foods providing K, Mg, folate, Ca, vitamins A and C, and soluble fibre as well as an array of bioactive substances including carotenoids and flavonoids^(^^4^^–^^6^^)^. Nutritional guidance encourages consumption of fruit as part of a balanced and healthy diet, although guidance often recommends limited consumption of fruit in the form of juice citing concerns over a lack of fibre and the potential for excessive energy intake^(^^7^^)^. Juices also tend to have moderately high-glycaemic index ratings^(^^8^^)^, indicating a relatively rapid and high post-prandial glucose response as compared with foods with a lower glycaemic index, and diets lower in these types of simple carbohydrates may be relevant for the prevention and management of some chronic diseases including T2D^(^^9^^)^. However, results from *in vitro* and animal studies suggest that polyphenols may favourably affect glucose–insulin homeostasis through a variety of mechanisms^(^^10^^)^. The net effect of 100 % fruit juice on glucose metabolism and biomarkers of diabetes therefore reflects a complex interplay of numerous factors.

Results from human studies of associations between 100 % fruit juice consumption and risk of T2D or effects on diabetes biomarkers have been conflicting. A recent meta-analysis of thirteen prospective cohorts reported no association between consumption of fruit juice and incident T2D^(^^11^^)^. When further adjusted for obesity, the meta-analysis showed a 7 % increased risk for incident T2D, though the presence of significant heterogeneity limits the quality of this evidence^(^^11^^)^.

No meta-analysis of randomised controlled trials (RCT) with a specific focus on 100 % fruit juices has been identified. Wang *et al.*^(^^12^^)^ reported finding no significant effect on glycaemic control and blood insulin in a meta-analysis of twelve RCT collectively described as 100 % fruit juice. The analysis included studies using juice products other than 100 % fruit juice^(^^12^^)^, namely beverages prepared from freeze-dried whole fruit which provided a substantial source of dietary fibre^(^^13^^,^^14^^)^, beverages prepared from fruit juice sweetened using no-energy sweeteners^(^^15^^,^^16^^)^, and a study in which both the test and control beverages were prepared from 100 % fruit juice^(^^17^^)^. The identified meta-analysis therefore was not exclusively based on RCT of 100 % fruit juice compared with a non-juice control. Several years have passed since completion of the meta-analysis and recent clinical trials examining the effects of 100 % fruit juice consumption on glucose–insulin homeostasis provide further insight into the role of juice on biomarkers of diabetes risk.

The purpose of the present study was to systematically review the literature to identify RCT in which effects of 100 % fruit juice on measures of glucose control and insulin sensitivity have been examined and, based on the totality of evidence, to re-evaluate in a meta-analysis the effects of 100 % fruit juice on these biomarkers for diabetes risk.

## Methods

### Literature search and study selection

The present systematic review and meta-analysis followed the Preferred Reporting Items for Systematic Reviews and Meta-Analyses (PRISMA) statement^(^^18^^)^. A comprehensive literature search was conducted to identify clinical studies examining the relationship between the consumption of 100 % fruit juice and measures of glucose–insulin homeostasis, with no exclusions based on age, ethnicity or health status of the study population. A pre-specified protocol is not available for this study. The search of PubMed was conducted with terms (juice[TIAB] OR juices[TIAB]) AND (‘controlled trial’ OR ‘clinical trial’ OR crossover OR cross-over OR random*) with no limits other than English-language papers. The search of EMBASE was conducted using similar terms. Terms for specific measures of glucose control or insulin sensitivity were not included in the search string to allow for identification of studies in which measures of interest were collected as secondary outcomes or in the course of routine patient monitoring. The initial search was conducted on 23 March 2015, and searches were updated on 14 April 2016. Supplemental literature searches included review of reference lists in relevant studies and pertinent review articles and correspondence with researchers in the field.

The search results were screened independently by two investigators (M. M. M., E. C. B.) for eligible studies and discrepancies were resolved by consensus. Clinical trials were eligible if the following criteria were met: (1) the trial was randomised and conducted in human subjects; (2) the trial was a controlled intervention providing 100 % fruit juice and a control beverage (e.g. sugar/carbohydrate or energy-matched beverage, water, or no beverage); (3) the fruit juice consumed was identified as 100 % fruit juice (including single-strength juice prepared from concentrate or a blend of 100 % fruit juices) or any juice specified by name such as, but not limited to, apple, blueberry, cherry, cranberry, grape, grapefruit, orange, pear, pomegranate or strawberry juice; (4) subjects consumed 100 % fruit juice for a minimum of 2 weeks; (5) outcome data for at least one measure of glucose control or insulin sensitivity were reported; and (6) reported outcomes included change from baseline values or baseline and endpoint values with error terms. Tomato juice was not included as a fruit juice in this analysis as it is typically marketed as a vegetable juice.

### Data extraction

All eligible studies were reviewed and pertinent data were extracted, including: name of the first author, publication year, study design, geographic location of the intervention, demographic and health characteristics of the study population (e.g. age, sex, presence of obesity or a chronic disease, BMI), sample size, intervention duration, juice type, a description of the control product, and outcomes measured. Additional data extracted from the studies included sugar content of the 100 % fruit juice and control beverages, volume of juice consumed per d, baseline fasting blood glucose level, information on randomisation, double-blinding, and withdrawals and dropouts to develop quality scores of each study based on the Jadad criteria^(^^19^^)^. Information on funding was reviewed to determine if the research was completed with support from industry funding, including donation of study product. Data extraction was completed independently by one investigator and reviewed for accuracy by another (M. M. M. and E. C. B. or K. A. B.). The extracted information included that provided by study investigators in response to requests for missing data and study details^(^^20^^–^^23^^)^. Fasting blood glucose, fasting blood insulin, the homeostatic model assessment of insulin resistance (HOMA-IR), glycosylated Hb (HbA1c) and other reported measures of glucose–insulin homeostasis (oral glucose tolerance test, insulinogenic and Matsuda indices) were captured as change from baseline or as baseline and post-intervention values. Fasting glucose values were converted to mmol/l assuming 1 mg/dl = 0·05551 mmol/l. Fasting insulin values were converted to pmol/l assuming 1 µU/ml = 6·0 pmol/l^(^^24^^)^.

### Statistical analysis

A meta-analysis was conducted to quantify the effects of consumption of 100 % fruit juice on each measure of glucose control or insulin sensitivity reported in at least three studies. The meta-analysis evaluated the mean difference from baseline and end of treatment values between the 100 % juice treatment and control group of fasting blood glucose, fasting blood insulin, HOMA-IR and HbA1c. The *I*^2^ statistic was used to assess statistical heterogeneity between studies.

For studies not reporting the change from baseline to the end of intervention in both the treatment and control groups, the mean difference was calculated as (Treatment_end_ − Treatment_baseline_) − (Control_end_ − Control_baseline_). The method described by Curtin *et al.*^(^^25^^)^ was used to estimate the mean difference and associated standard error from the combined parallel and cross-over studies. A pooled estimate of the variance of the mean difference was estimated for each study. For parallel studies, the estimate was derived using the reported estimates of the standard errors or the calculated standard errors as derived from the reported CI estimates. For cross-over studies when estimates of the standard deviation (or standard error) were only available for baseline and post-intervention measurements, the pooled variance was estimated using standard errors for the intervention and control groups and an imputed correlation coefficient of 0·5^(^^26^^,^^27^^)^ following the approach described by Higgins & Green^(^^27^^)^. The correlation coefficient (0·5) assumed in the derivation was similar to the average coefficient derived from a study in which variances were provided for baseline, treatment end, and change from baseline measures of fasting blood glucose and HOMA-IR; the correlation coefficient for fasting blood insulin derived from this study was 0·7^(^^28^^)^. Hence, a sensitivity analysis assuming a correlation coefficient of 0·7 was conducted for fasting blood insulin.

A random-effects model was used to determine the mean and 95 % CI of differences in changes from baseline between 100 % juice and control groups. The approach described in DerSimonian & Laird^(^^29^^)^ was used to conduct the random-effects model. Stratified analyses by study characteristics were planned *a priori* to investigate sources of heterogeneity and univariate meta-regressions were conducted for further investigation. For each of the biomarkers of diabetes, analyses were conducted for type of fruit juice (apple, berry, blend, citrus, grape, pomegranate), type of control beverage (beverage matched for carbohydrate or sugar and/or energy, beverage without carbohydrate or sugar and/or energy, no beverage), volume of intervention beverage (as reported in the original studies for the meta-regression analysis, and categorised as ≤250 ml/d, >250 ml/d in the stratified analysis), duration of intervention (as reported in the original studies for the meta-regression analysis, and categorised as 2–7 weeks, ≥8 weeks in the stratified analysis), baseline fasting blood glucose (<5·6 mmol/l, ≥5·6 mmol/l), location of study (North America, Europe, Asia), study design (parallel, cross-over), outcome (primary, secondary) and Jadad quality score (<4, ≥4). Industry funding (with or without) was also examined in the stratified analysis. Multivariate meta-regressions including all factors with statistically significant (*P* < 0·05) regression coefficients were planned for subsequent analyses.

Publication bias was assessed for outcomes with more than ten studies by visual examination of funnel plots of the standard error of the mean difference *v.* the mean difference and the Egger's regression asymmetry test^(^^30^^)^. Sensitivity analyses to explore the influence of a single study on each meta-analysis were conducted by computing the meta-analysis estimates, omitting one study at a time. Level of significance was defined as *P* < 0·05. All statistical analyses were completed with STATA, version 12.1 (StataCorp LLC).

## Results

### Literature search, study characteristics

**Fig. 1.**
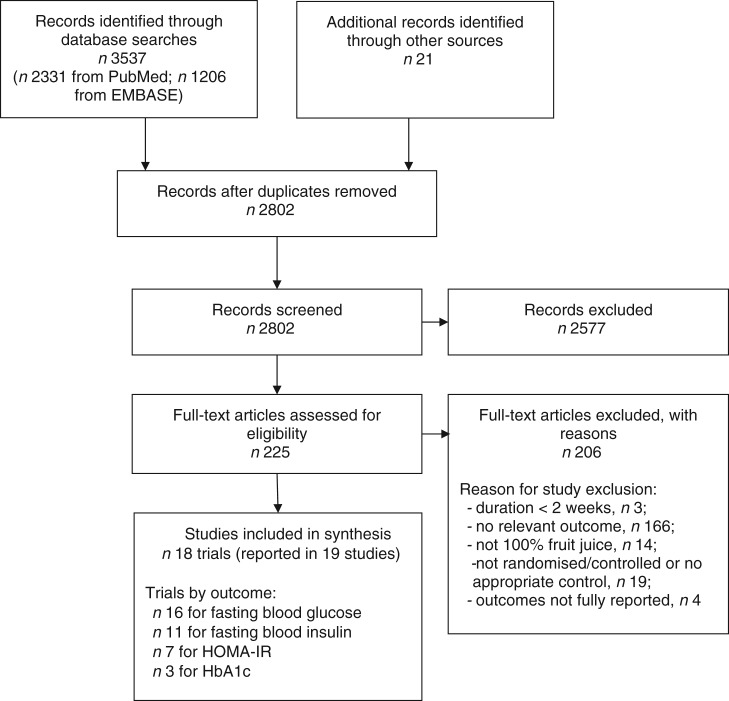
Flow diagram of study selection process. HOMA-IR, homeostatic model assessment of insulin resistance; HbA1c, glycosylated Hb.

A total of eighteen RCT of 100 % fruit juice were eligible for inclusion in the present review (Fig. 1). Characteristics of the eighteen RCT of 100 % fruit juice and measures of glucose control or insulin sensitivity included in the meta-analysis are presented in Table 1^(^^31^^)^. Two publications provided relevant outcomes from the same clinical trial and results from both publications were captured for the analysis^(^^28^^,^^32^^)^. In two of the identified studies, results of two comparisons of potential interest were presented^(^^33^^,^^34^^)^, though in order to avoid double-counting of studies, one comparison from each was selected for inclusion in the meta-analysis^(^^33^^)^. In a parallel-design study of the effects of Concord grape juice, the energy-matched control beverage (*v.* a no-beverage control) was selected for the control group^(^^33^^)^. In a cross-over study providing clear or cloudy apple juice within two of the five study arms^(^^34^^)^, clear apple juice was selected for the test group as this type of juice is assumed to be more commonly consumed.

**Table 1.** Characteristics of eighteen randomised controlled trials of 100 % fruit juice included in analysis

Reference	Location: continent (country)	Study population: health, sex, mean age (years) ± sd	Study design	Mean baseline BMI (kg/m^2^) ± sd	Subjects: test/control (*n*)	Mean baseline glucose (mmol/l)	Duration*	Juice test product†: type (ml/d), sugars (g/d)	Control product	Outcome(s), primary or secondary	Industry funding‡
Banini *et al.* (2006)^(^^35^^)^	North America (USA)	Healthy; M/F Test: 50 ± 13 Control: 56 ± 7·5	Parallel	Test: 29·3 ± 4·0 Control: 27·5 ± 5·4	8/15	<5·6	4 weeks	Grape (muscadine) 150, about 23 (estimated)	No beverage	FBG/FBI/HbA1c, P	Yes
Cerdá *et al.* (2006)^(^^20^^)^	Europe (Spain)	COPD; M Test: 60 ± 10·9 Control: 63·4 ± 8·9	Parallel	Test: 31·4 ± 4·8 Control: 30·6 ± 5·8	15/15	≥5·6	5 weeks	Pomegranate 400, about 53 (estimated)	Sugar-free bev	FBG, S	No
Codoñer-Franch *et al.* (2010)^(^^36^^)^	Europe (Spain)	Obese; M/F Test: 11·8 ± 2·4 Control: 12·1 ± 1·9	Parallel	Test: 29·3 ± 3·5 Control: 28·7 ± 4·2	20/20	<5·6	4 weeks	Mandarin 500, 53	No beverage	FBG/FBI/HOMA-IR, S	Yes
Dohadwala *et al.* (2010)^(^^37^^)^	North America (USA)	HTN/pre-HTN; M/F Test: 41 ± 13 Control: 44 ± 11	Cross-over	Test: 28 ± 3·8 Control: 28 ± 3·9	64/64	<5·6	8 weeks	Grape (Concord) 595§, 97	Sugar bev	FBG/FBI/HOMA-IR, S	Yes
González-Ortiz *et al.* (2011)^(^^21^^)^	North America (Mexico)	Obese; F Test: 36·3 ± 8·3 Control: 38·3 ± 10·4	Parallel	Test: 35·2 ± 3·1 Control: 33·8 ± 4·1	10/10	<5·6	1 month	Pomegranate 120, about 16 (estimated)	Sugar-free bev	FBG/FBI/other, P	No
Guo *et al.* (2014)^(^^38^^)^	Asia (China)	NAFLD; M/F 21·2 ± 1·2	Cross-over	Test: 25·3 ± 2·2 Control: 25·6 ± 2·5	44/43	<5·6	4 weeks	Bayberry 500, 46	Sugar bev	FBG, S	No
Habauzit *et al.* (2015)^(^^39^^)^	Europe (France)	Healthy; F 57·8 ± 3·7	Cross-over	25·7 ± 2·3	48/48	<5·6	6 months	Grapefruit (white) 340, 27	Sugar bev	FBG/FBI/HOMA-IR, S	Yes
Hollis *et al.* (2009)^(^^33^^)^	North America (USA)	Overweight; M/F Test: 22 ± 4 Control: 26 ± 9	Parallel	Test: 27·0 ± 1·6 Control: 27·0 ± 1·5	25/26	<5·6	12 weeks	Grape (Concord) 480, 82	Sugar bev	FBG, S	Yes
Krikorian *et al.* (2012)^(^^40^^)^	North America (USA)	MCI; M/F 76·9 ± 6·1	Parallel	Not reported	10/11	≥5·6	16 weeks	Grape (Concord) 532§, about 80 (estimated)	Sugar bev	FBG/FBI, S	Yes
Morand *et al.* (2011)^(^^41^^)^	Europe (France)	Overweight; M 56 ± 4·8	Cross-over	27·4 ± 1·4	23/23	<5·6	4 weeks	Orange 500, 45	Sugar bev	FBG, FBI, S	Yes
Ravn-Haren *et al.* (2013)^(^^34^^)^	Europe (Denmark)	Healthy; M/F 36·2 ± 17·9	Cross-over	22·3 ± 2·6	23/23	<5·6	4 weeks	Apple (clear) 500, 63	No beverage	FBI, S	No
Shidfar *et al.* (2012)^(^^22^^)^	Asia (Iran)	T2D; M 54·8 ± 9·1	Parallel	28·8 ± 3·9	29/29	≥5·6	12 weeks	Cranberry 240, about 31 (estimated)	Sugar-free bev	FBG, P	No
Silver *et al.* (2011)^(^^42^^)^	North America (USA)	Obese; M/F Test: 39·8 ± 8·4 Control: 38·7 ± 8·8	Parallel	Test: 35·2 ± 3·1 Control: 35·7 ± 3·5	22/23	<5·6	12 weeks	Grapefruit (white) about 360 (estimated), about 28 (estimated)	Sugar-free bev	FBG/FBI/HOMA-IR, S	Yes
Simpson *et al.*2016^(^^44^^)^	Europe (UK)	Overweight/obese, hypercholesterolaemic; M Test: 48·3 ± 3·9 Control: 48·9 ± 4·3	Parallel	Test: 29·9 ± 2·3 Control: 29·3 ± 1·7	18/18	<5·6	12 weeks	Orange 250, 22	Sugar bev	HOMA-IR, S	Yes
Sohrab *et al.* (2014, 2015)^(^^28^^,^^32^^)^	Asia (Iran)	T2D; M/F Test: 55 ± 6·7 Control: 56·9 ± 6·8	Parallel	Test: 29·4 ± 3·9 Control: 28·6 ± 4·2	22/22	≥5·6	12 weeks	Pomegranate 250, 24	Sugar bev	FBG/FBI/HOMA-IR/HbA1c, S	No
Sumner *et al.* (2005)^(^^45^^)^	North America (USA)	CHD; M/F Test: 69 ± 11 Control: 69 ± 9	Parallel	Test: 28 ± 6 Control: 29 ± 5	25/18	≥5·6	3 months	Pomegranate 240, about 32 (estimated)	Sugar bev	FBG/HbA1c, S	Yes
Tjelle *et al.* (2015)^(^^23^^)^	Europe (Norway)	HTN/pre-HTN; M/F Test: 61 ± 6 Control: 62 ± 6	Parallel	Test: 26 ± 3 Control: 26 ± 3	47/43	<5·6	12 weeks	Blend (red grape, chokeberry, cherry, bilberry) 500, 66	Sugar bev	FBG, S	Yes
Tsang *et al.* (2012)^(^^43^^)^	Europe (UK)	Overweight/obese; M/F 50·4 ± 6·1	Cross-over	26·8 ± 3·3	28/28	<5·6	4 weeks	Pomegranate 500, about 66 (estimated)	Sugar bev	FBG/FBI/HOMA-IR, P	Yes

M, male; F, female; FBG, fasting blood glucose; FBI, fasting blood insulin; HbA1c, glycosylated Hb; P, primary; COPD, chronic obstructive pulmonary disease; sugar-free bev, beverage without carbohydrate or sugar and/or energy; S, secondary; HOMA-IR, homeostatic model assessment of insulin resistance; HTN, hypertension; sugar bev, beverage matched for carbohydrate or sugar and/or energy; NAFLD, non-alcoholic fatty liver disease; MCI, mild cognitive impairment; T2D, type 2 diabetes.

* Duration = length of dietary intervention for each test or control period.

† When not reported, the amount of sugar provided by the test product was estimated using data from the United States Department of Agriculture National Nutrient Database for the specific type of fruit juice tested^(^^31^^)^.

‡ Industry funding includes financial support and/or test products.

§ Juice volume calculated assuming mean body weight of study participants.

Sixteen of the eighteen included trials reported data for fasting blood glucose^(^^20^^–^^23^^,^^28^^,^^32^^,^^33^^,^^35^^–^^43^^)^, eleven reported fasting blood insulin^(^^21^^,^^28^^,^^34^^–^^37^^,^^39^^–^^43^^)^, seven reported HOMA-IR^(^^28^^,^^36^^,^^37^^,^^39^^,^^42^^–^^44^^)^ and three reported HbA1c^(^^28^^,^^35^^,^^45^^)^. Duration of supplementation ranged from 2 weeks to 6 months, with the majority of studies providing the intervention for a period of 8 weeks or more. The studies were predominantly conducted in Europe (*n* 8) or North America (*n* 7), with the remaining three studies conducted in Asia (*n* 3).

All but five studies included a test and control arm; four studies included three arms^(^^23^^,^^33^^,^^41^^,^^42^^)^ and one study included five arms^(^^34^^)^. Although all studies were controlled, the type of control beverage provided varied across studies. Across the eighteen studies, twelve indicated some level of industry funding including financial support and/or test products.

Six studies had a Jadad score of ≥4^(^^23^^,^^28^^,^^32^^,^^33^^,^^37^^,^^39^^,^^45^^)^, while the remaining trials (twelve of eighteen) had a Jadad score of <4. As shown in Table 2, each study was identified by the investigators as a randomised study, though only five provided the methods of random sequence generation. Eleven of the studies were reported to be double-blind, and eight of these detailed a method regarded as appropriate within the Jadad criteria. The remaining eight studies were open label or single blind. Descriptions of study withdrawals, including both the number and reason, were provided in fourteen of the eighteen studies. No studies described inappropriate randomisation or double-blinding methods. High risk for attrition bias was identified in three studies and a cross-over study with an unspecified washout period between treatments presented unclear risk for bias.

**Table 2.** Jadad scores of study quality and major sources of potential bias by study

Reference	Randomised		Doubled-blind		Withdrawals and dropouts		Major sources of potential bias
	Yes/no*	Method†	Yes/no*	Method†	Description	Cumulative Jadad score	
Banini *et al.* (2006)^(^^35^^)^	1	0	0	0	0	1	Unclear risk of selection bias, attrition bias; high risk of performance bias
Cerdá *et al.* (2006)^(^^20^^)^	1	0	1	1	0	3	Unclear risk of selection bias, attrition bias
Codoñer-Franch *et al.* (2010)^(^^36^^)^	1	0	0	0	0	1	Unclear risk of selection bias, attrition bias; high risk of performance bias
Dohadwala *et al.* (2010)^(^^37^^)^	1	1	1	1	1	5	High risk of attrition bias – 23 % of subjects withdrew from the study
González-Ortiz *et al.* (2011)^(^^21^^)^	1	0	1	0	1	3	Unclear risk of selection bias, performance bias
Guo *et al.* (2014)^(^^38^^)^	1	0	1	0	1	3	Unclear risk of selection bias, performance bias
Habauzit *et al.* (2015)^(^^39^^)^	1	0	1	1	1	4	Unclear risk of selection bias
Hollis *et al.* (2009)^(^^33^^)^	1	0	1	1	1	4	Unclear risk of selection bias
Krikorian *et al.* (2012)^(^^40^^)^	1	0	1	1	0	3	Unclear risk of selection bias, attrition bias
Morand *et al.* (2011)^(^^41^^)^	1	1	0	0	1	3	High risk of performance bias
Ravn-Haren *et al.* (2013)^(^^34^^)^	1	0	0	0	1	2	Unclear risk of selection bias; high risk of performance bias; high risk of attrition bias – 32% of enrolled subjects dropped out before completing all treatments; unclear risk of study design bias – unspecified washout period between treatments
Shidfar *et al.* (2012)^(^^22^^)^	1	0	1	0	1	3	Unclear risk of selection bias, performance bias
Silver *et al.* (2011)^(^^42^^)^	1	1	0	0	1	3	High risk of performance bias; high risk of attrition bias – 20 % of study subjects dropped out between study weeks 6 and 9
Simpson *et al.* (2016)^(^^44^^)^	1	1	0	0	1	3	High risk of performance bias
Sohrab *et al.* (2014, 2015)^(^^28^^,^^32^^)^	1	0	1	1	1	4	Unclear risk of selection bias
Sumner *et al.* (2005)^(^^45^^)^	1	1	1	1	1	5	–
Tjelle *et al.* (2015)^(^^23^^)^	1	0	1	1	1	4	Unclear risk of selection bias
Tsang *et al.* (2012)^(^^43^^)^	1	0	0	0	1	2	Unclear risk of selection bias; high risk of performance bias

* Yes, 1; no, 0.

† Appropriate, 1; inappropriate, −1; not specified, 0.

### Effect of 100 % fruit juice on fasting blood glucose

**Fig. 2.**
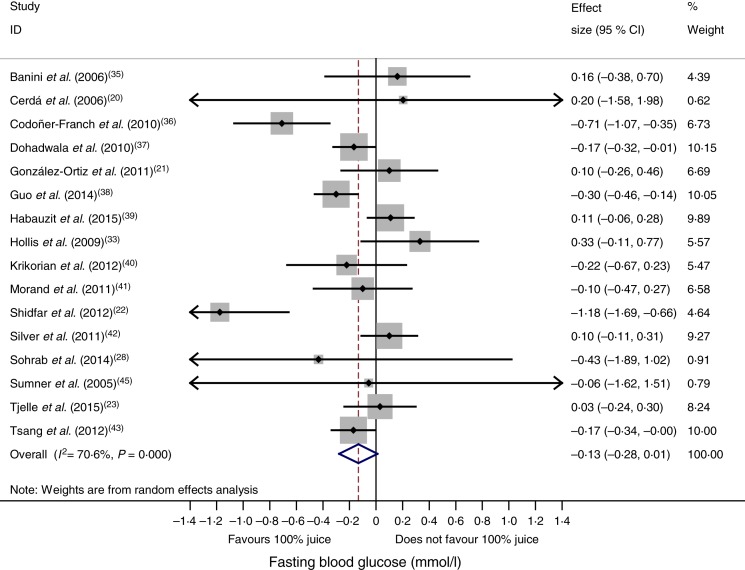
Meta-analysis of the effects of 100 % fruit juice on fasting blood glucose. Square values represent the mean difference of fasting blood glucose values (mmol/l) based on a random-effects model; 95 % confidence intervals are represented by horizontal lines. Square size is proportional to the weight of each study. The diamond represents the pooled estimate of mean differences (*P* = 0·07).

In the meta-analysis of fasting blood glucose data reported in sixteen RCT, consumption of 100 % fruit juice had no significant effect on fasting blood glucose compared with the control treatment (−0·13 (95 % CI −0·28, 0·01) mmol/l; *P* = 0·07) (Fig. 2); there was moderate to high heterogeneity among the studies (*P* < 0·01, *I*^2^ = 70·6). Stratified analyses (Table 3) used to evaluate potential sources of heterogeneity resulted in mean difference estimates ranging from −0·70 to 0·20. Associations that were statistically significant in stratified analyses included an intervention of 2–7 weeks (−0·20 (95 % CI −0·38, −0·08) mmol/l; *P* = 0·03, *I*^2^ = 57·0), and studies with a Jadad score of <4 (−0·23 (95 % CI −0·43, −0·03) mmol/l; *P* = 0·03, *I*^2^ = 74·8); in both of these stratified analyses, fasting blood glucose was lowered by consumption of 100 % fruit juice compared with the control group. The only factor with a statistically significant regression coefficient in the univariate meta-regressions was study location; therefore no multivariate meta-regression analyses were conducted.

**Table 3.** Stratified analyses of effects of 100 % fruit juice on fasting blood glucose (FBG)

					Test of heterogeneity		Unadjusted (univariate) meta-regression		
Parameter	Studies (*n*)	Net change	95 % CI	Overall *P*	*P*	*I*^2^ (%)	RC	se	*P*
All studies	16	−0·13	−0·28, 0·01	0·07	<0·005	70·6			
Fruit juice type									
Apple	–	–	–	–	–	–	–	–	–
Berry	2	−0·70	−1·56, 0·15	0·11	<0·005	90·0	−0·56	0·32	0·11
Blend	1	0·03	−0·24, 0·30	0·83	–	–	0·10	0·39	0·81
Citrus	4	−0·12	−0·43, 0·19	0·45	<0·005	83·1	−0·06	0·27	0·84
Grape	4	−0·03	−0·28, 0·22	0·83	0·14	45·9	0·07	0·28	0·82
Pomegranate	5	−0·12	−0·27, 0·03	0·10	0·72	0	Reference	–	–
Control group									
Sugar bev	10	−0·09	−0·21, 0·03	0·14	0·04	49·6	0·26	0·29	0·39
Sugar-free bev	4	−0·24	−0·84, 0·36	0·43	<0·005	85·6	0·13	0·34	0·71
No beverage	2	−0·30	−1·15, 0·55	0·49	0·01	85·5	Reference	–	–
Volume of juice							<0·005	<0·005	0·75
≤250 ml/d	5	−0·29	−0·93, 0·36	0·38	<0·005	77·9	–	–	–
>250 ml/d	11	−0·11	−0·25, 0·03	0·13	<0·005	69·1	–	–	–
Duration							0·01	0·01	0·44
2–7 weeks	7	−0·20	−0·38, −0·02	0·03	0·03	57·0	–	–	–-
≥8 weeks	9	−0·09	−0·30, 0·12	0·39	<0·005	72·9	–	–	–
Baseline FBG									
<5·6 mmol/l	11	−0·08	−0·21, 0·06	0·28	<0·005	70·7	Reference	–	–
≥5·6 mmol/l	5	−0·51	−1·10, 0·09	0·10	0·07	54·3	−0·49	0·23	0·06
Location									
Asia	3	−0·65	−1·36, 0·05	0·07	0·01	80·0	Reference	–	–
Europe	6	−0·14	−0·36, 0·09	0·24	<0·005	72·8	0·45	0·24	0·08
North America	7	0·01	−0·15, 0·16	0·94	0·19	31·0	0·62	0·24	0·02
Outcome									
Primary	4	−0·25	−0·70, 0·20	0·27	<0·005	83·4	Reference	–	–
Secondary	12	−0·10	−0·26, 0·05	0·20	<0·005	65·6	0·14	0·21	0·53
Study design									
Cross-over	5	−0·13	−0·28, 0·02	0·08	0·02	66·9	0·02	0·19	0·91
Parallel	11	−0·15	−0·43, 0·12	0·27	<0·005	73·9	Reference	–	–
Jadad score									
<4	10	−0·23	−0·43, -0·03	0·03	<0·005	74·8	Reference	–	–
≥4	6	0·02	−0·15, 0·18	0·86	0·13	40·9	0·26	0·18	0·17
Study funding									
Industry support	11	−0·07	−0·21, 0·07	0·34	<0·005	62·5	0·30	0·20	0·17
No industry support	5	−0·38	−0·84, 0·08	0·11	<0·005	75·1	Reference	–	–

RC, regression coefficient; sugar bev, beverage matched for carbohydrate or sugar and/or energy; sugar-free bev, beverage with non-energy-containing or no added sweetener.

### Effect of 100 % fruit juice on fasting blood insulin

**Fig. 3.**
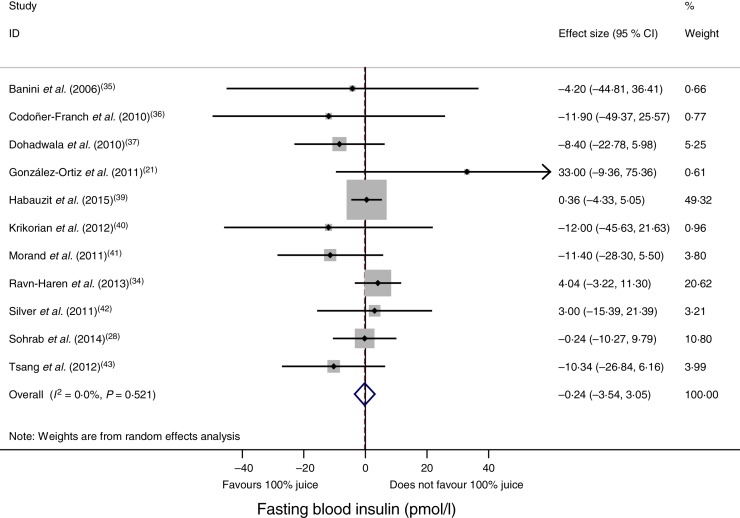
Meta-analysis of the effects of 100 % fruit juice on fasting blood insulin. Square values represent the mean difference of fasting blood insulin values (pmol/l) based on a random-effects model; 95 % confidence intervals are represented by horizontal lines. Square size is proportional to the weight of each study. The diamond represents the pooled estimate of mean differences (*P* = 0·89).

Compared with the control treatment, consumption of 100 % fruit juice had no significant effect on fasting blood insulin (−0·24 (95 % CI −3·54, 3·05) pmol/l; *P* = 0·89) with no heterogeneity (*P* = 0·52, *I*^2^ = 0) in the eleven RCT included in the analysis (Fig. 3). Stratified analyses of the effects of 100 % fruit juice on fasting blood insulin resulted in mean difference estimates ranging from −8·50 to 11·6, none of which was statistically significant (Table 4).

**Table 4.** Stratified analyses of effects of 100 % fruit juice on fasting blood insulin

					Test of heterogeneity		Unadjusted (univariate) meta-regression		
Parameter	Studies (*n*)	Net change	95 % CI	Overall *P*	*P*	*I*^2^ (%)	RC	se	*P*
All studies	11	−0·24	−3·54, 3·05	0·89	0·52	0			
Fruit juice type									
Apple	1	4·04	−3·22, 11·3	0·28	.	–	5·59	5·66	0·36
Berry	–	–	–	–	–	–	–	–	–
Blend	–	–	–	–	–	–	–	–	–
Citrus	4	−0·44	−4·80, 3·92	0·84	0·53	0	1·11	4·83	0·83
Grape	3	−8·50	−21·07, 4·07	0·19	0·96	0	−6·95	7·71	0·40
Pomegranate	3	−0·72	−14·90, 13·46	0·92	0·16	46·1	Reference	–	–
Control group									
Sugar bev	6	−1·69	−5·52, 2·14	0·39	0·50	0	−5·52	4·78	0·28
Sugar-free bev	2	11·68	−14·98, 38·34	0·39	0·20	38·3	4·95	9·73	0·63
No beverage	3	3·24	−3·78, 10·25	0·37	0·67	0	Reference	–	–
Volume of juice							−0·02	0·02	0·26
≤250 ml/d	3	2·66	−11·29, 16·61	0·71	0·32	13·5	–	–	–
>250 ml/d	8	−0·44	−3·96, 3·07	0·81	0·46	0	–	–	–
Duration							0·09	0·24	0·73
2–7 weeks	6	−2·13	−11·60, 7·35	0·66	0·21	30·6	–	–	–
≥8 weeks	5	−0·44	−4·40, 3·51	0·83	0·76	0	–	–	–
Baseline FBG									
<5·6 mmol/l	9	−0·41	−4·48, 3·66	0·84	0·37	7·4	Reference	–	–
≥5·6 mmol/l	2	−1·20	−10·81, 8·41	0·81	0·51	0	−1·09	5·24	0·84
Location									
Asia	1	−0·24	−10·27, 9·79	0·96	–	–	Reference	–	–
Europe	5	−0·43	−5·24, 4·38	0·86	0·30	17·6	0·33	5·74	0·96
North America	5	−2·70	−12·78, 7·39	0·60	0·41	0	−2·45	7·63	0·76
Outcome									
Primary	3	0·73	−23·20, 24·65	0·95	0·17	42·7	Reference	–	–
Secondary	8	0	−3·39, 3·39	1·00	0·63	0	4·58	7·54	0·56
Study design									
Cross-over	5	−1·41	−6·58, 3·76	0·59	0·22	30·4	−0·54	4·50	0·91
Parallel	6	0·21	−7·78, 8·20	0·96	0·65	0	Reference	–	–
Jadad score									
<4	8	−0·89	−7·60, 5·81	0·79	0·35	10·2	Reference	–	–
≥4	3	−0·44	−4·52, 3·63	0·83	0·53	0	−0·48	3·86	0·90
Study funding									
Industry support	8	−1·84	−5·83, 2·16	0·37	0·68	0	−6·13	4·31	0·19
No industry support	3	3·20	−3·78, 10·19	0·37	0·30	16·8	Reference	–	–

RC, regression coefficient; sugar bev, beverage matched for carbohydrate or sugar and/or energy; sugar-free bev, beverage with non-energy-containing or no added sweetener; FBG, fasting blood glucose.

### Effect of 100 % fruit juice on insulin resistance

**Fig. 4.**
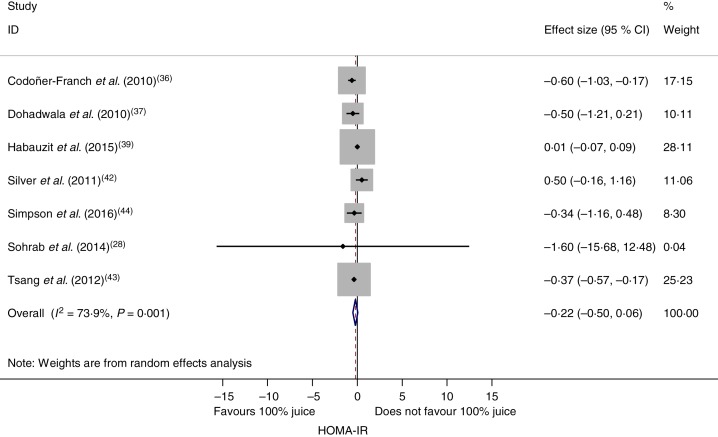
Meta-analysis of the effects of 100 % fruit juice on the homeostatic model assessment of insulin resistance (HOMA-IR). Square values represent the mean difference of the HOMA-IR index based on a random-effects model; 95 % confidence intervals are represented by horizontal lines. Square size is proportional to the weight of each study. The diamond represents the pooled estimate of mean differences (*P* = 0·13).

The effect of 100 % fruit juice on HOMA-IR was not significant (−0·22 (95 % CI −0·50, 0·06); *P* = 0·13) with moderate to high heterogeneity (*P* < 0·01, *I*^2^ = 73·9) (Fig. 4). Stratified analyses resulted in mean difference estimates ranging from −1·60 to 0·50 (Table 5). In stratified analyses, HOMA-IR was significantly lower in the 100 % fruit juice groups compared with the control group in studies in which pomegranate juice was consumed (−0·37 (95 % CI −0·57, −0·18); *P* < 0·005, *I*^2^ = 0) or intervention duration was 2–7 weeks (−0·41 (95 % CI −0·59, −0·23); *P* < 0·005, *I*^2^ = 0). In the univariate meta-regressions, only volume of juice intervention showed a statistically significant inverse association with change in HOMA-IR (regression coefficient = −0·002; *P* = 0·01).

**Table 5.** Stratified analyses of effects of 100 % fruit juice on the homeostatic model assessment of insulin resistance

					Test of heterogeneity		Unadjusted (univariate) meta-regression		
Parameter	Studies (*n*)	Net change	95 % CI	Overall *P*	*P*	*I*^2^ (%)	RC	se	*P*
All studies	7	−0·22	−0·5, 0·06	0·13	<0·005	73·9			
Fruit juice type									
Apple	–	–	–	–	–	–	–	–	–
Berry	–	–	–	–	–	–	–	–	–
Blend	–	–	–	–	–	–	–	–	–
Citrus	4	−0·11	−0·52, 0·29	0·58	0·02	70·8	0·26	0·44	0·58
Grape	1	−0·50	−1·21, 0·21	0·17	–	–	−0·12	0·64	0·86
Pomegranate	2	−0·37	−0·57, −0·18	0·00	0·86	0	Reference	–	–
Control group									
Sugar bev	5	−0·23	−0·53, 0·07	0·14	0·01	71·9	0·38	0·34	0·33
Sugar-free bev	1	0·50	−0·16, 1·16	0·14	–	–	1·10	0·51	0·10
No beverage	1	−0·60	−1·03, −0·17	0·01	–	–	Reference	–	–
Volume of juice							<0·005	<0·005	0·01
≤ 250 ml/d	2	−0·34	−1·17, 0·48	0·41	0·86	0	–	–	–
>250 ml/d	5	−0·21	−0·51, 0·10	0·19	<0·005	82·2	–	–	–
Duration							0·02	0·01	0·08
2–7 weeks	2	−0·41	−0·59, −0·23	0·00	0·35	0	–	–	–
≥8 weeks	5	−0·01	−0·25, 0·23	0·92	0·31	16·9		–	–
Baseline FBG									
<5·6 mmol/l	6	−0·22	−0·50, 0·07	0·14	<0·005	78·2	Reference	–	–
≥5·6 mmol/l	1	−1·60	−15·68, 12·48	0·82	–	–	−1·38	7·36	0·86
Location									
Asia	1	−1·60	−15·68, 12·48	0·82	–	–	Reference	–	–
Europe	4	−0·28	−0·61, 0·04	0·08	<0·005	83·9	1·31	7·31	0·87
North America	2	0·01	−0·97, 0·99	0·99	0·04	75·4	1·62	7·31	0·84
Outcome									
Primary	1	−0·37	−0·57, −0·18	<0·005	–	–	Reference	–	–
Secondary	6	−0·17	−0·52, 0·18	0·34	0·03	58·8	0·20	0·39	0·64
Study design									
Cross-over	3	−0·22	−0·56, 0·12	0·20	<0·005	85·4	−0·05	0·35	0·90
Parallel	4	−0·18	−0·83, 0·48	0·60	0·06	59·9	Reference	–	–
Jadad score									
<4	4	−0·27	−0·64, 0·10	0·16	0·05	60·8	Reference	–	–
≥4	3	0	−0·08, 0·09	0·95	0·37	0	0·11	0·34	0·75
Study funding									
Industry support	6	−0·22	−0·50, 0·07	0·14	<0·005	78·2	1·38	7·36	0·86
No industry support	1	−1·60	−15·68, 12·48	0·82	–	–	Reference	–	–

RC, regression coefficient; sugar bev, beverage matched for carbohydrate or sugar and/or energy; sugar-free bev, beverage with non-energy-containing or no added sweetener; FBG, fasting blood glucose.

### Effect of 100 % fruit juice on glycosylated Hb

**Fig. 5.**
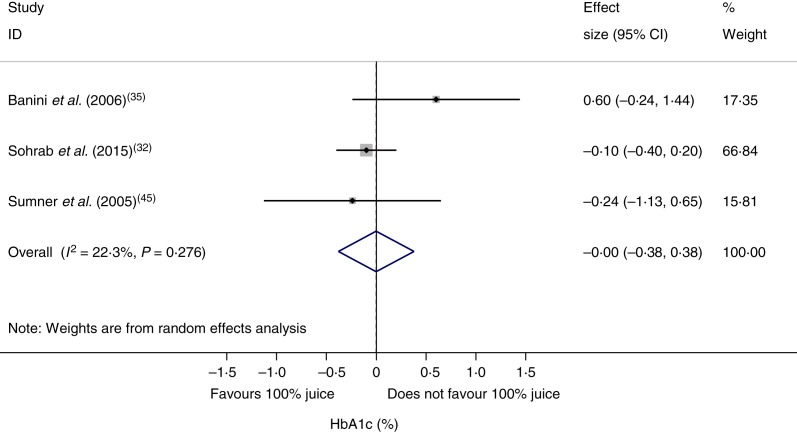
Meta-analysis of the effects of 100 % fruit juice on glycosylated Hb (HbA1c; %). Square values represent the mean difference of HbA1c values based on a random-effects model; 95 % confidence intervals are represented by horizontal lines. Square size is proportional to the weight of each study. The diamond represents the pooled estimate of mean differences (*P* = 1·00).

The effect of 100 % fruit juice on HbA1c was not significant (−0·001 (95 % CI −0·38, 0·38) %; *P* > 0·99) with low heterogeneity (*P* < 0·01, *I*^2^ = 22·3) (Fig. 5). Stratified analyses resulted in mean difference estimates ranging from −0·11 to 0·60 (Table 6). No statistically significant associations were observed in the stratified analyses or univariate meta-regressions.

**Table 6.** Stratified analyses of effects of 100 % fruit juice on glycosylated Hb (%)

					Test of heterogeneity		Unadjusted (univariate) meta-regression		
Parameter	Studies (*n*)	Net change	95 % CI	Overall *P*	*P*	*I*^2^ (%)	RC	se	*P*
All studies	3	−0·001	−0·38, 0·38	1·00	0·28	22·3	–	–	–
Fruit juice type									
Apple	–	–	–	–	–	–	–	–	–
Berry	–	–	–	–	–	–	–	–	–
Blend	–	–	–	–	–	–	–	–	–
Citrus	–	–	–	–	–	–	–	–	–
Grape	1	0·60	−0·24, 1·44	0·16	–	–	0·71	0·45	0·36
Pomegranate	2	−0·11	−0·40, 0·17	0·43	0·77	0	Reference	–	–
Control group									
Sugar bev	2	−0·11	−0·40, 0·17	0·43	0·77	0	−0·71	0·45	0·36
Sugar-free bev	–	–	–	–	–	–	–	–	–
No beverage	1	0·60	−0·24, 1·44	0·16	–	–	Reference	–	–
Volume of juice							−0·01	0·01	0·37
≤250 ml/d	3	0	−0·38, 0·38	1·00	0·28	22·3	–	–	–
>250 ml/d	–	–	–	–	–	–			
Duration							−0·09	0·06	0·36
2–7 weeks	1	0·60	−0·24, 1·44	0·16	–	–	–	–	–
≥8 weeks	2	−0·11	−0·40, 0·17	0·43	0·77	0	–	–	–
Baseline FBG									
<5·6 mmol/l	1	0·60	−0·24, 1·44	0·16	–	–	Reference	–	–
≥5·6 mmol/l	2	−0·11	−0·40, 0·17	0·43	0·77	0	−0·71	0·45	0·36
Location									
Asia	1	−0·10	−0·40, 0·20	0·52	–	–	Reference	–	–
Europe	–	–	–	–	–	–	–	–	–
North America	2	0·19	−0·63, 1·02	0·65	0·18	44·8	0·29	0·60	0·71
Outcome									
Primary	1	0·60	−0·24, 1·44	0·16	–	–	Reference	–	–
Secondary	2	−0·11	−0·40, 0·17	0·43	0·77	0	−0·71	0·45	0·36
Study design									
Cross-over	–	–	–	–	–	–	–	–	–
Parallel	3	0	−0·38, 0·38	1·00	0·28	22·3	Reference	–	–
Jadad score									
<4	1	0·60	−0·24, 1·44	0·16	–	–	Reference	–	–
≥4	2	−0·11	−0·40, 0·17	0·43	0·77	0	−0·71	0·45	0·36
Study funding									
Industry support	2	0·19	−0·63, 1·02	0·65	0·18	44·8	0·29	0·60	0·71
No industry support	1	−0·10	−0·40, 0·20	0·52	–	–	Reference	–	–

RC, regression coefficient; sugar bev, beverage matched for carbohydrate or sugar and/or energy; sugar-free bev, beverage with non-energy-containing or no added sweetener; FBG, fasting blood glucose.

### Publication bias

**Fig. 6.**
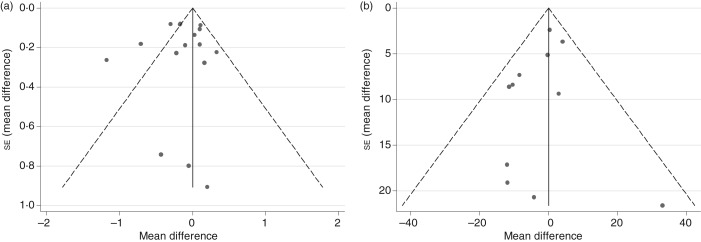
Funnel plots with pseudo 95 % confidence limits for detection of publication bias among randomised controlled trials examining fasting blood glucose (a) and fasting blood insulin (b). For fasting blood glucose, *P* value for Egger's test = 0·80. For fasting blood insulin, *P* value for Egger's test = 0·38.

The potential for publication bias was investigated through visual inspection of funnel plots for analyses with a sufficient number of studies, namely fasting blood glucose and fasting blood insulin. Visual inspection of funnel plots for fasting blood glucose showed that all but three studies^(^^22^^,^^36^^,^^38^^)^ fell inside the funnel. These three studies had relatively smaller mean difference estimates indicative of statistically significant beneficial effects of the intervention beverages. Sensitivity analyses in which each study was removed from the analysis individually or all three simultaneously resulted in a mean difference that was still negative and not significantly different from zero (data not shown), thus suggesting no publication bias. Results from the Egger's test supported this conclusion for both fasting blood glucose and insulin (Fig. 6; *P* = 0·80 and 0·38, respectively).

### Sensitivity analysis

Sensitivity analyses that excluded individual RCT resulted in estimates similar to values derived from all studies, although some mean differences became statistically significant. In the case of fasting blood glucose, omission of either of three studies^(^^33^^,^^39^^,^^42^^)^ resulted in overall mean difference estimates that were statistically significant and indicative of a beneficial effect of 100 % fruit juice consumption (mean difference of −0·16; 95 % CI −0·31, −0·005). The effects of 100 % fruit juice on fasting blood insulin or HOMA-IR were not found to be sensitive to any particular study included in the meta-analysis. The sensitivity analysis conducted for fasting blood insulin assuming a correlation coefficient of 0·7 in the derivation of the pooled variance for cross-over studies showed no significant effect of 100 % fruit juice on fasting blood insulin (results not shown).

## Discussion

The present systematic review and meta-analysis provides a comprehensive, quantitative assessment of the relationship between 100 % fruit juice and measures of glycaemic control. Results from this meta-analysis of eighteen RCT show no significant effect of 100 % fruit juice on fasting blood glucose, fasting blood insulin, insulin resistance as evaluated by HOMA-IR or HbA1c. Additionally, results of stratified analyses confirm the lack of consistent associations between 100 % fruit juice consumption and these markers for diabetes. The absence of any clear adverse or beneficial effect of 100 % fruit juice on markers of diabetes in the present meta-analysis of available RCT suggests a largely neutral role of 100 % fruit juice on glucose–insulin homeostasis.

The findings of the present meta-analysis are generally consistent with a 2014 meta-analysis by Wang *et al*.^(^^12^^)^. Overall, seven studies were included in both the previous and the present meta-analysis^(^^20^^,^^21^^,^^33^^,^^35^^,^^37^^,^^41^^,^^45^^)^. The eleven additional trials included in the present analysis reflect recent additions to the literature as well as studies not captured by Wang *et al*. ^(^^12^^)^. As in the present analysis, no significant effect on glycaemic control measured by fasting glucose, fasting insulin or HbA1c was reported in the meta-analysis of studies identified as 100 % fruit juice by Wang *et al.*^(^^12^^)^. In contrast to the present analysis in which no effect of 100 % fruit juice on HOMA-IR was found, a significant increase in HOMA-IR was reported by Wang *et al.*^(^^12^^)^ based on data from three trials, only one of which was considered eligible for the present meta-analysis^(^^45^^)^. The other two HOMA-IR values in the meta-analysis by Wang *et al.*^(^^12^^)^ include a value from a study not meeting our inclusion criteria^(^^16^^)^, and a value attributed to the trial by Sumner *et al.*^(^^45^^)^ which was neither reported in that paper nor noted as sourced from the study authors. The value attributed to Sumner accounted for 78 % of the weighted mean HOMA-IR in the meta-analysis by Wang *et al*.^(^^12^^)^, and therefore largely explains the difference between the previous and present analyses. Findings from the present meta-analysis of RCT on markers for diabetes are also consistent with findings from a meta-analysis of prospective cohorts showing that consumption of 100 % fruit juice is not associated with increased risk of T2D^(^^46^^)^ and findings from a more recent meta-analysis of cohorts in which diabetes was clinically verified^(^^11^^)^.

Stratified analyses were conducted as part of the present meta-analysis to investigate specific conditions that may adversely or beneficially affect glucose–insulin homeostasis. The stratifications included parameters to differentiate among the juice intervention, study population and study characteristics. Consistent with the primary findings, results from the stratified analyses showed no significant effect of 100 % fruit juice on fasting blood insulin and HbA1c, though results of both analyses, and in particular HbA1c, were limited by the small number of studies. Stratified analyses of fasting blood glucose showed a significantly greater reduction from baseline with 100 % fruit juice compared with the control in trials with an intervention duration of 2–7 weeks and trials with a Jadad score <4. The significant effect observed in shorter studies could reflect higher compliance by study participants over a shorter study intervention. However, a cross-tabulation of the studies with fasting blood glucose data by these two factors reveals that all studies with a shorter duration of intervention (2–7 weeks) also had a Jadad score <4, which is an indicator of a lower methodological quality trial. As detailed in the review of Jadad scores by studies (Table 2), many of the RCT suggesting a beneficial effect of 100 % fruit juice on fasting blood glucose had one or more major sources of potential bias, and in particular high or unclear performance bias. Findings in the stratified analyses therefore provide some though limited support for a beneficial effect of fruit juice on fasting blood glucose. Stratified analyses of HOMA-IR also showed a significantly greater reduction from baseline with 100 % fruit juice compared with the control in trials with an intervention duration of 2–7 weeks, and additionally in trials with pomegranate juice. The significance of these findings also is limited, as each analysis was based on data from only two studies and did not notably reduce heterogeneity. The inverse association of volume of 100 % fruit juice with HOMA-IR identified in univariate meta-regressions also is an indicator of potentially beneficial effects of 100 % fruit juice, albeit very small as evidenced by the relatively small regression coefficient.

A variety of juices was provided as test beverages across the studies and the types and concentrations of some bioactives in fruit juice vary across fruit juice type. For example, pomegranate juice is a unique source of ellagitannins, including punicalagins^(^^47^^)^. Among citrus juices, grapefruit juice is a concentrated source of naringin while the primary flavonoid in orange juice is hesperidin^(^^48^^,^^49^^)^. Cranberries are a source of polyphenols including procyanidins, anthocyanins, quercetin and myricitrin^(^^50^^)^, the primary anthocyanins in many grape juices are glucosides of cyanidin or delphinidin^(^^49^^,^^51^^)^, and apple juice is a source of quercetin^(^^49^^,^^51^^)^. The amount of fruit juice consumed during the dietary interventions was also highly variable, with juice intake of approximately two cups or more (480 to 595 ml) per d in nine of the interventions and one cup or less in six studies (<250 ml). The amount of sugars provided by the juices ranged from 22 to 97 g. The ‘100 % fruit juice’ category therefore represents a heterogeneous food group and differences in the type and composition of fruit juices may be important when considering the physiological effects of fruit juice, including effects on glycaemic control and insulin sensitivity. Stratified analyses, however, showed no clear differences across categorisations and heterogeneity was not notably reduced.

The RCT of 100 % fruit juices identified in the present review also used a variety of control products, including energy-containing beverages matched for sugar or carbohydrate content, sugar-free beverages containing non-energy-containing sweeteners or only water, and, in some studies, no beverage. Peluso & Palmery^(^^52^^)^ have noted that selection of an appropriate placebo in studies of the postprandial response to fruit juice is critical, with energy and sugar-matched beverages (including proportions of glucose and fructose) providing an appropriate control to assess the effects of bioactives in juice, and a water beverage providing an appropriate control to assess the effects of juice as a whole^(^^52^^)^. Stratified analyses of fasting blood glucose by these control group comparisons did not result in significant effects, suggesting that neither the non-sugar juice components nor the juice itself had an effect on fasting blood glucose in the clinical trials.

The effects of 100 % fruit juice on measures of glucose control or insulin sensitivity varied little across study population characteristics including baseline fasting blood glucose levels. Body weight status of study participants is another important characteristic as overweight and obesity are recognised risk factors for the development of T2D due to decreased sensitivity of non-adipose tissue to insulin^(^^2^^)^. In all but one of the studies with reported BMI data^(^^34^^)^, mean BMI exceeded 25 kg/m^2^, indicating that the study populations were generally overweight or obese individuals. The available reported data on body weight or BMI indicate no differences in change between the juice intervention and control groups. In addition to weight status, the study populations also represented a variety of health states including healthy, diabetic, hypertensive/pre-hypertensive, hypercholesterolaemic, and adults with various other conditions. While many of these conditions are common co-morbidities of overweight and obesity, it is difficult to delineate their independent role in the relationship between 100 % fruit juice consumption and diabetes risk.

Many methods are used to assess bias in RCT when conducting a meta-analysis, including the Jadad criteria^(^^53^^)^. Based on the Jadad criteria, which reflect randomisation, double blinding, and recording of withdrawals and dropouts^(^^19^^)^, the majority of studies in this meta-analysis had an unclear risk of selection bias due to absence of a description of the random sequence generation. A relatively high proportion of studies were non-double-blind (seven of eighteen) which points to a high risk of performance bias. Regarding attrition bias, the majority of studies (fourteen of eighteen) provided information on participant disposition throughout the study and the data largely indicate low risk of bias, though attrition was relatively high (20–32 %) in three studies^(^^34^^,^^37^^,^^42^^)^ and the remaining studies have unknown or potentially high risk for this source of bias. Other potential sources of bias not captured in the Jadad criteria but noted upon review of the studies include unknown or potentially high bias due to an unspecified washout period between interventions^(^^34^^)^.

Despite concerns that 100 % fruit juice may have adverse effects on glycaemic control, primarily as a result of sugars in these beverages, results from this analysis of RCT do not support a conclusion that 100 % fruit juice adversely affects glucose–insulin homeostasis. The results largely indicate no effect on glycaemic control. Some stratified analyses suggest the possibility of a beneficial effect of 100 % fruit juice on fasting blood glucose and HOMA-IR, though the evidence is inconclusive based on limitations of the available data. A mechanism by which 100 % fruit juice may have a favourable effect on measures of glycaemic control is not clearly known, though some evidence, largely from *in vitro* and animal studies, indicates that polyphenols may favourably affect glucose–insulin homeostasis through a variety of mechanisms including inhibition of glucose absorption, stimulation of insulin secretion from the pancreas and change in glucose release from the liver, activation of insulin receptors and glucose uptake by cells, and modulation of cell signalling pathways and gene expression^(^^10^^)^. Further research is needed to understand these effects.

A strength of the present meta-analysis is the large number of randomised, controlled clinical trials identified for inclusion in the quantitative analysis. The broad though focused search strategy identified studies in which glucose metabolism outcomes were primary outcomes as well as studies in which the measures were collected as secondary outcomes or routine monitoring during the clinical trials. The large number of identified studies allowed for stratified analyses by similar characteristics of the intervention, study population, and overall study design to further explore effects of 100 % fruit juice on diabetes biomarkers. However, sample sizes in many stratified analyses were relatively small, and many analyses may result in detection of spurious associations, therefore these findings must be interpreted with caution.

Although the total number of identified studies was relatively large, variability among some parameters of study interventions, populations, and overall study design are limitations of this analysis and must be considered when interpreting the findings. The included studies reflect a diverse range of juice interventions (both type of juice and amount consumed), intervention durations, variable dietary restrictions throughout the intervention period (and typically no restrictions on consumption of other types of 100 % fruit juice), study populations with a range of health conditions, and studies with potential for some bias. Variability in these factors and potentially other factors not considered in the analysis contribute to the observed heterogeneity among studies, thus making it difficult to conclusively interpret the findings.

### Conclusion

In conclusion, the available RCT indicate that repeated intake of 100 % fruit juice does not have a significant effect on glycaemic control or measures of insulin resistance. These findings from RCT of markers for diabetes are consistent with findings from some observational studies suggesting that consumption of 100 % fruit juice is neutral regarding risk of T2D. Results from stratified analyses and univariate meta-regressions also largely showed no significant associations between 100 % fruit juice and these measures of glucose control. High-quality studies of glucose–insulin homeostasis measures monitored in well-defined and controlled populations are needed to further clarify the effects of 100 % fruit juice on diabetes risk as evaluated by these biomarkers. Such research focused on commonly consumed juices served in moderate daily portions reflective of prudent dietary guidance would provide important information to further our understanding of the role of 100 % fruit juice on glycaemic control.

## References

[1] Rolic G (2016) WHO global report on diabetes: a summary. Int J Non-Commun Dis 1, 3–8.

[2] American Diabetes Association (2016) Classification and diagnosis of diabetes. Sec. 2. In Standards of Medical Care in Diabetes-2016. *Diabetes Care* **39**, Suppl. 1, S13–S22. 10.2337/dc16-S005 26696675

[3] Mozaffarian D (2016) Dietary and policy priorities for cardiovascular disease, diabetes, and obesity: a comprehensive review. Circulation 133, 187–225. 26746178 10.1161/CIRCULATIONAHA.115.018585 PMC4814348

[4] Li BW, Andrews KG & Pehrsson PR (2002) Individual sugars, soluble, and insoluble dietary fiber contents of 70 high consumption foods. J Food Comp Anal 15, 715–723.

[5] Liu RH (2013) Health-promoting components of fruits and vegetables in the diet. Adv Nutr 4, 384s–392s. 23674808 10.3945/an.112.003517 PMC3650511

[6] Rampersaud GC (2007) A comparison of nutrient density scores for 100% fruit juices. J Food Sci 72, S261–S266. 17995788 10.1111/j.1750-3841.2007.00324.x

[7] United States Department of Health and Human Services & United States Department of Agriculture (2015) Dietary Guidelines for Americans 2015-2020, 8th edition. http://health.gov/dietaryguidelines/2015/guidelines/(accessed November 2017).

[8] Atkinson FS, Foster-Powell K & Brand-Miller JC (2008) International tables of glycemic index and glycemic load values: 2008. Diabetes Care 31, 2281–2283. 18835944 10.2337/dc08-1239 PMC2584181

[9] Augustin LS, Kendall CW, Jenkins DJ, (2015) Glycemic index, glycemic load and glycemic response: an International Scientific Consensus Summit from the International Carbohydrate Quality Consortium (ICQC). Nutr Metab Cardiovasc Dis 25, 795–815. 26160327 10.1016/j.numecd.2015.05.005

[10] Hanhineva K, Torronen R, Bondia-Pons I, (2010) Impact of dietary polyphenols on carbohydrate metabolism. Int J Mol Sci 11, 1365–1402. 20480025 10.3390/ijms11041365 PMC2871121

[11] Imamura F, O'Connor L, Ye Z, (2015) Consumption of sugar sweetened beverages, artificially sweetened beverages, and fruit juice and incidence of type 2 diabetes: systematic review, meta-analysis, and estimation of population attributable fraction. BMJ 351, h3576. 26199070 10.1136/bmj.h3576 PMC4510779

[12] Wang B, Liu K, Mi M, (2014) Effect of fruit juice on glucose control and insulin sensitivity in adults: a meta-analysis of 12 randomized controlled trials. PLOS ONE 9, e95323. 24743260 10.1371/journal.pone.0095323 PMC3990696

[13] Basu A, Fu DX, Wilkinson M, (2010) Strawberries decrease atherosclerotic markers in subjects with metabolic syndrome. Nutr Res 30, 462–469. 20797478 10.1016/j.nutres.2010.06.016 PMC2929388

[14] Basu A, Du M, Leyva MJ, (2010) Blueberries decrease cardiovascular risk factors in obese men and women with metabolic syndrome. J Nutr 140, 1582–1587. 20660279 10.3945/jn.110.124701 PMC2924596

[15] Basu A, Betts NM, Ortiz J, (2011) Low-energy cranberry juice decreases lipid oxidation and increases plasma antioxidant capacity in women with metabolic syndrome. Nutr Res 31, 190–196. 21481712 10.1016/j.nutres.2011.02.003 PMC3075541

[16] Dohadwala MM, Holbrook M, Hamburg NM, (2011) Effects of cranberry juice consumption on vascular function in patients with coronary artery disease. Am J Clin Nutr 93, 934–940. 21411615 10.3945/ajcn.110.004242 PMC3076649

[17] Reshef N, Hayari Y, Goren C, (2005) Antihypertensive effect of sweetie fruit in patients with stage I hypertension. Am J Hypertens 18, 1360–1363. 16202862 10.1016/j.amjhyper.2005.05.021

[18] Moher D, Aliberati A, Tetzlaff J, (2009) Preferred Reporting Items for Systematic Reviews and Meta-Analyses: The PRISMA statement. PLOS Medicine 6, e1000097. 19621072 10.1371/journal.pmed.1000097 PMC2707599

[19] Jadad AR, Moore RA, Carroll D, (1996) Assessing the quality of reports of randomized clinical trials: is blinding necessary? Control Clin Trials 17, 1–12. 8721797 10.1016/0197-2456(95)00134-4

[20] Cerdá B, Soto C, Albaladejo MD, (2006) Pomegranate juice supplementation in chronic obstructive pulmonary disease: a 5-week randomized, double-blind, placebo-controlled trial. Eur J Clin Nutr 60, 245–253. 16278692 10.1038/sj.ejcn.1602309

[21] González-Ortiz M, Martínez-Abundis E, Espinel-Bermúdez MC, (2011) Effect of pomegranate juice on insulin secretion and sensitivity in patients with obesity. Ann Nutr Metab 58, 220–223. 21811060 10.1159/000330116

[22] Shidfar F, Heydari I, Hajimiresmaiel SJ, (2012) The effects of cranberry juice on serum glucose, apoB, apoA-I, Lp(a), and paraoxonase-1 activity in type 2 diabetic male patients. J Res Med Sci 17, 355–360. 23267397 PMC3526129

[23] Tjelle TE, Holtung L, Bohn SK, (2015) Polyphenol-rich juices reduce blood pressure measures in a randomised controlled trial in high normal and hypertensive volunteers. Br J Nutr 114, 1054–1063. 26227795 10.1017/S0007114515000562

[24] Marcovina S, Bowsher RR, Miller WG, (2007) Standardization of insulin immunoassays: report of the American Diabetes Association Workgroup. Clin Chem 53, 711–716. 17272483 10.1373/clinchem.2006.082214

[25] Curtin F, Altman DG & Elbourne D (2002) Meta-analysis combining parallel and cross-over clinical trials. I: continuous outcomes. Stat Med 21, 2131–2144. 12210629 10.1002/sim.1205

[26] Follmann D, Elliott P, Suh I, (1992) Variance imputation for overviews of clinical trials with continuous response. J Clin Epidemiol 45, 769–773. 1619456 10.1016/0895-4356(92)90054-q

[27] Higgins JPT & Green S (editors) (2011) Cochrane Handbook for Systematic Reviews of Interventions, version 5.1.0 [updated March 2011]. The Cochrane Collaboration. http://handbook.cochrane.org

[28] Sohrab G, Nasrollahzadeh J, Zand H, (2014) Effects of pomegranate juice consumption on inflammatory markers in patients with type 2 diabetes: a randomized, placebo-controlled trial. J Res Med Sci 19, 215–220. 24949028 PMC4061642

[29] DerSimonian R & Laird N (1986) Meta-analysis in clinical trials. Control Clin Trials 7, 177–188. 3802833 10.1016/0197-2456(86)90046-2

[30] Egger M, Davey Smith G, Schneider M, (1997) Bias in meta-analysis detected by a simple, graphical test. BMJ 315, 629–634. 9310563 10.1136/bmj.315.7109.629 PMC2127453

[31] United States Department of Agriculture Agricultural Research Service, Nutrient Data Laboratory (2016) USDA National Nutrient Database for Standard Reference, Release 28 (slightly revised). http://www.ars.usda.gov/ba/bhnrc/ndl

[32] Sohrab G, Angoorani P, Tohidi M, (2015) Pomegranate (*Punicagranatum*) juice decreases lipid peroxidation, but has no effect on plasma advanced glycated end-products in adults with type 2 diabetes: a randomized double-blind clinical trial. Food Nutr Res 59, 28551. 26355954 10.3402/fnr.v59.28551 PMC4565065

[33] Hollis JH, Houchins JA, Blumberg JB, (2009) Effects of Concord grape juice on appetite, diet, body weight, lipid profile, and antioxidant status of adults. J Am Coll Nutr 28, 574–582. 20439553 10.1080/07315724.2009.10719789

[34] Ravn-Haren G, Dragsted LO, Buch-Andersen T, (2013) Intake of whole apples or clear apple juice has contrasting effects on plasma lipids in healthy volunteers. Eur J Nutr 52, 1875–1889. 23271615 10.1007/s00394-012-0489-z

[35] Banini AE, Boyd LC, Allen JC, (2006) Muscadine grape products intake, diet and blood constituents of non-diabetic and type 2 diabetic subjects. Nutrition 22, 1137–1145. 17030113 10.1016/j.nut.2006.08.012

[36] Codoñer-Franch P, López-Jaén AB, De La Mano-Hernández A, (2010) Oxidative markers in children with severe obesity following low-calorie diets supplemented with mandarin juice. Acta Paediatr 99, 1841–1846. 20528796 10.1111/j.1651-2227.2010.01903.x

[37] Dohadwala MM, Hamburg NM, Holbrook M, (2010) Effects of Concord grape juice on ambulatory blood pressure in prehypertension and stage 1 hypertension. Am J Clin Nutr 92, 1052–1059. 20844075 10.3945/ajcn.2010.29905 PMC2954442

[38] Guo H, Zhong R, Liu Y, (2014) Effects of bayberry juice on inflammatory and apoptotic markers in young adults with features of non-alcoholic fatty liver disease. Nutrition 30, 198–203. 24377455 10.1016/j.nut.2013.07.023

[39] Habauzit V, Verny MA, Milenkovic D, (2015) Flavanones protect from arterial stiffness in postmenopausal women consuming grapefruit juice for 6 mo: a randomized, controlled, crossover trial. Am J Clin Nutr 102, 66–74. 26016866 10.3945/ajcn.114.104646

[40] Krikorian R, Boespflug EL, Fleck DE, (2012) Concord grape juice supplementation and neurocognitive function in human aging. J Agric Food Chem 60, 5736–5742. 22468945 10.1021/jf300277g

[41] Morand C, Dubray C, Milenkovic D, (2011) Hesperidin contributes to the vascular protective effects of orange juice: a randomized crossover study in healthy volunteers. Am J Clin Nutr 93, 73–80. 21068346 10.3945/ajcn.110.004945

[42] Silver HJ, Dietrich MS & Niswender KD (2011) Effects of grapefruit, grapefruit juice and water preloads on energy balance, weight loss, body composition, and cardiometabolic risk in free-living obese adults. Nutr Metab 8, 8. 10.1186/1743-7075-8-8 PMC3039556 21288350

[43] Tsang C, Smail NF, Almoosawi S, (2012) Intake of polyphenol-rich pomegranate pure juice influences urinary glucocorticoids, blood pressure and homeostasis model assessment of insulin resistance in human volunteers. J Nutr Sci 1, e9. 25191556 10.1017/jns.2012.10 PMC4153032

[44] Simpson EJ, Mendis B & Macdonald IA (2016) Orange juice consumption and its effect on blood lipid profile and indices of the metabolic syndrome; a randomised, controlled trial in an at-risk population. Food Funct 7, 1884–1891. 26965492 10.1039/c6fo00039h

[45] Sumner MD, Elliott-Eller M, Weidner G, (2005) Effects of pomegranate juice consumption on myocardial perfusion in patients with coronary heart disease. Am J Cardiol 96, 810–814. 16169367 10.1016/j.amjcard.2005.05.026

[46] Xi B, Li S, Liu Z, (2014) Intake of fruit juice and incidence of type 2 diabetes: a systematic review and meta-analysis. PLOS ONE 9, e93471. 24682091 10.1371/journal.pone.0093471 PMC3969361

[47] Tzulker R, Glazer I, Bar-Ilan I, (2007) Antioxidant activity, polyphenol content, and related compounds in different fruit juices and homogenates prepared from 29 different pomegranate accessions. J Agric Food Chem 55, 9559–9570. 17914875 10.1021/jf071413n

[48] Nogata Y, Sakamoto K, Shiratsuchi H, (2006) Flavonoid composition of fruit tissues of citrus species. Biosci Biotechnol Biochem 70, 178–192. 16428836 10.1271/bbb.70.178

[49] Bhagwat S, Haytowitz DB & Holden JM (2014) USDA Database for the Flavonoid Content of Selected Foods, release 3.1, May 2014 update. US Department of Agriculture, Agricultural Research Service. http://www.ars.usda.gov/nutrientdata/flav

[50] Vvedenskaya IO, Rosen RT, Guido JE, (2004) Characterization of flavonols in cranberry (*Vaccinium macrocarpon*) powder. J Agric Food Chem 52, 188–195. 14733493 10.1021/jf034970s

[51] Xu Y, Simon JE, Welch C, (2011) Survey of polyphenol constituents in grapes and grape-derived products. J Agric Food Chem 59, 10586–10593. 21879745 10.1021/jf202438d

[52] Peluso I & Palmery M (2014) Risks of misinterpretation in the evaluation of the effect of fruit-based drinks in postprandial studies. Gastroenterol Res Pract 2014, 870547. 25610461 10.1155/2014/870547 PMC4295616

[53] Berger VW & Alperson SY (2009) A general framework for the evaluation of clinical trial quality. Rev Recent Clin Trials 4, 79–88. 19463104 10.2174/157488709788186021 PMC2694951

